# Comparative Phytochemical Profiling and Biological Activities of Two *Asplenium ceterach* L. Extracts

**DOI:** 10.3390/molecules31172978

**Published:** 2026-08-25

**Authors:** Marijana Skorić, Milica Milutinović, Uroš Gašić, Marija Ivanov, Đura Nakarada, Miloš Mojović, Ana Ćirić, Nevenka Gligorijević, Đurđa Ivković, Petar Ristivojević, Ladislav Luc, Suzana Živković

**Affiliations:** 1 Institute for Biological Research “Siniša Stanković”-National Institute of the Republic of Serbia, University of Belgrade, Bulevar despota Stefana 142, 11108 Belgrade, Serbia; milica.milutinovic@ibiss.bg.ac.rs (M.M.); marija.smiljkovic@ibiss.bg.ac.rs (M.I.); rancic@ibiss.bg.ac.rs (A.Ć.); ladislav.luc@ibiss.bg.ac.rs (L.L.); 2 Faculty of Physical Chemistry, University of Belgrade, Studentski trg 12-16, 11158 Belgrade, Serbia; djura@ffh.bg.ac.rs (Đ.N.); milos@ffh.bg.ac.rs (M.M.); 3 Department of Experimental Oncology, Institute for Oncology and Radiology of Serbia, Pasterova 14, 11000 Belgrade, Serbia; nevenka.malesevic@ncrc.ac.rs; 4 Innovative Centre of the Faculty of Chemistry Ltd., University of Belgrade, Studentski trg 12-16, 11158 Belgrade, Serbia; djurdja@chem.bg.ac.rs; 5 Faculty of Chemistry, University of Belgrade, Studentski trg 12-16, 11158 Belgrade, Serbia; ristivojevic@chem.bg.ac.rs

**Keywords:** rustyback fern, antimicrobial activity, antioxidant potential, *α*-amylase activity, cytotoxicity

## Abstract

Methanol (ME) and dichloromethane (DCM) extracts of rustyback fern (*Asplenium ceterach* L.) sporophytes were analyzed for antioxidant properties by thin-layer chromatography (TLC)-based bioautography, using DPPH as the detection reagent, and were comparatively tested for their antimicrobial, antibiofilm, and cytotoxic activities. The bioautography assay indicated that the rustyback fern ME extract had significant antioxidant potential and moderate *α*-amylase activity compared to the DCM extract. Both extracts exhibited pronounced scavenging activity towards hydroxyl radicals, as well as antibacterial and antifungal potential against all tested strains, although to varying degrees. The Gram-negative *Escherichia coli* and Gram-positive *Bacillus cereus* were the most sensitive bacteria, while the most sensitive fungus was the yeast *Candida krusei*. Furthermore, the tested extracts showed notable capability to prevent *Candida albicans* biofilm establishment, with no significant difference between the two extracts examined and an inhibition range of 72–74%. Cytotoxic screening indicated the safety of the extracts even at high concentrations, up to 100 μg mL^−1^. The results of the present study highlight *A. ceterach* extracts as a significant source of bioactive compounds, supporting their potential use as potent antioxidants and for combating diseases caused by multidrug-resistant microorganisms.

## 1. Introduction

Desiccation-tolerant (DT) plants, also known as resurrection plants, can survive extreme water loss (up to 95% of their relative water content) by temporarily ceasing most biological functions. After rehydration, they rapidly return to normal physiological activities in existing tissues without apparent long-term damage. Desiccation tolerance is found primarily in bryophytes and pteridophytes [1,2]. About 1% of ferns are considered DT as sporophytes [3,4,5,6]. Due to their ability to survive for months at extreme environmental margins, DT ferns are expected to contain many useful phytochemicals with specialized biological functions.

The resurrection fern, *Asplenium ceterach* L. (rustyback fern), is common in Europe, North Africa, and the Middle East, with scattered populations extending eastward to western China, typically found on base-rich rocks and mortar walls. The ethnomedicinal uses of *A. ceterach*, its chemical constituents, and pharmacological properties associated with its therapeutic potential have been extensively researched (reviewed in [7]). Numerous reports have documented the biological activity of *A. ceterach*, including antimicrobial, antioxidant, cytotoxic, anticancer, and diuretic activities; its anti-inflammatory properties; DNA damage protection potential; and hepatoprotective and hematoprotective properties [7]. Previous studies have demonstrated the presence of a wide range of specialized metabolites in extracts of *A. ceterach* sporophytes, including phenolic hydroxybenzoic and hydroxycinnamic acids, acetophenones, flavonoids, lignans, and xanthones, making them an important source of antioxidants [8].

Phenolic compounds are not distributed equally in plants and exhibit variable stability, making their extraction more challenging [9]. Due to the unstable nature of phenolic compounds, the extraction method is a crucial step in their isolation and subsequent compound identification [10]. Solvents play an important role in the extraction process, considerably affecting the amount and nature of the specialized metabolites of the plants. According to the literature, no solvent is universally accepted as the best for the extraction of polyphenols; nevertheless, it is generally believed that solvents with higher polarity are often most effective, owing to the high solubility of polyphenols in such solvents. For example, polar solvents are typically used to extract phenolic components and their glycosidic derivatives and saponins, etc., while fatty acids and steroids, etc., are extracted using non-polar solvents [11]. Numerous studies have reported the effect of solvents on the diversity of specialized metabolites and/or their biological properties. Therefore, to enhance the biological properties of phytocompounds, proper selection of the appropriate solvent is essential for the development of a universal method for the extraction of all phenolic compounds. Comparative biological studies of the same plant extract obtained using different solvents are beneficial in achieving this goal. However, there is no detailed report in the literature on the phytoconstituents of *A. ceterach* extracted using solvents with different polarities that includes comparisons of their biological activities. For this purpose, in the current study, the aerial parts of the plant were extracted using two different solvents: methanol (ME) and dichloromethane (DCM). Each extract of *A. ceterach* was analyzed separately to determine its chemical constituents and assess its biological properties. In this context, the objective of our study was to identify the main differences in the phytochemical profiles of *A. ceterach* extracts produced using different extraction solvents and to determine their biological activities. Expanding upon our preliminary findings [12], this study introduces, for the first time, an extensive high-performance thin-layer chromatography (HPTLC)-α-amylase assay while also providing a detailed evaluation of the antioxidant, antibiofilm, and cytotoxic potential of *A. ceterach* extracts.

## 2. Results

### 2.1. UHPLC-LTQ OrbiTrap MS Analysis of A. ceterach Extracts

**Figure 1:**
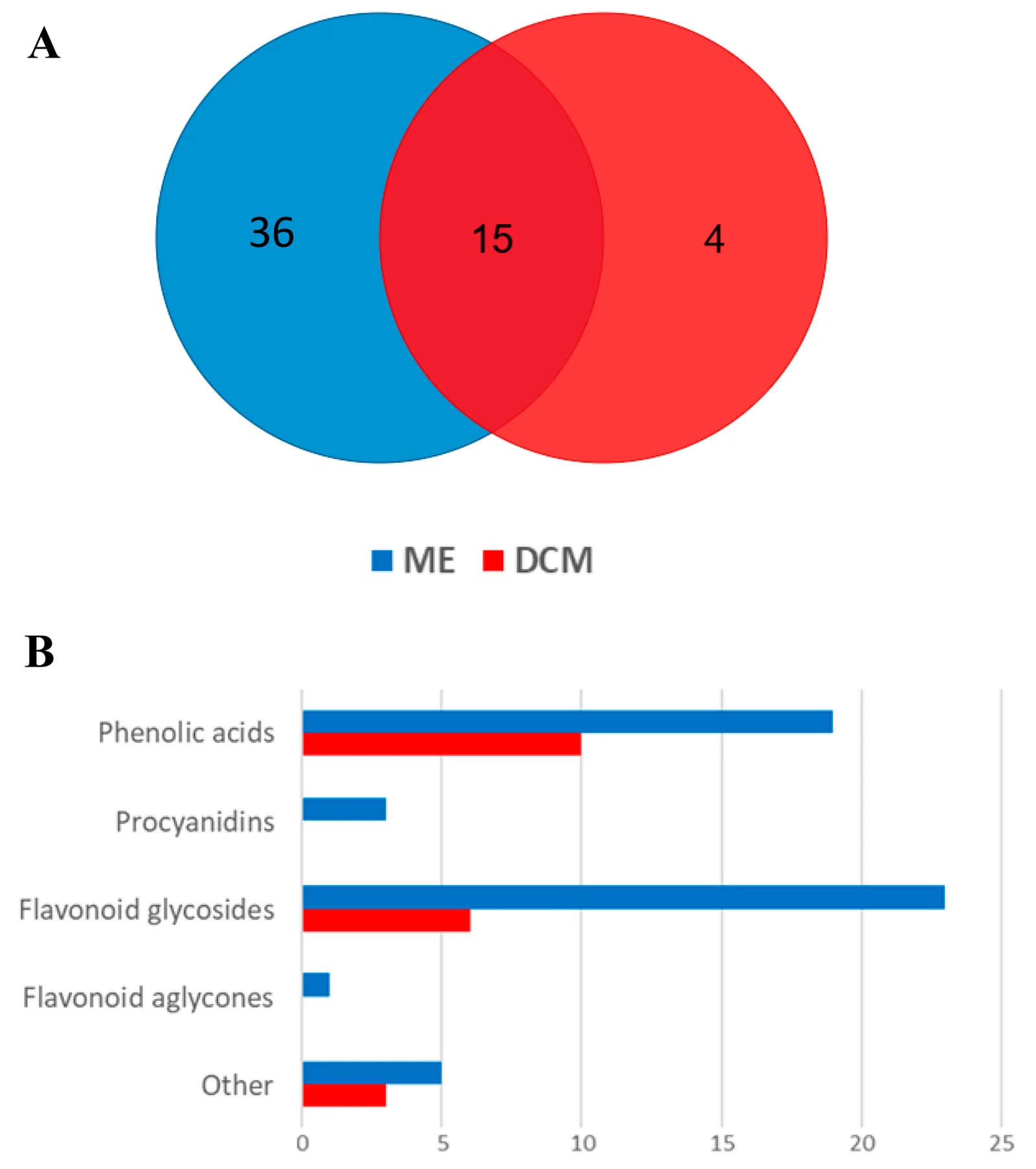
Comparative distribution of the identified compounds in methanol (ME) and dichloromethane (DCM) extracts of *A. ceterach* analyzed by UHPLC-LTQ OrbiTrap MS. (**A**) Venn diagram illustrating the number of unique and shared compounds between ME and DCM extracts (**B**) Total number of compounds identified in each major group of phenolics in the ME and DCM extracts. Red indicates dichloromethane (DCM) extract; blue indicates methanol (ME) extract.

Using the untargeted UHPLC–MS^4^ Orbitrap approach, a total of 55 metabolites were identified in two different extracts of *A. ceterach* (Table 1, Figure 1). Based on MS^2^, MS^3^, and MS^4^ fragmentation of molecules and the mass of the deprotonated molecule [M−H]^−^, various metabolites were detected in ME and DCM extracts. Metabolites are divided into several groups according to their structural characteristics (Table 1). Thus, the given table lists 21 phenolic acids and derivatives, 3 proanthocyanidins, 24 flavonoid glycosides, 1 flavonoid aglycone, and 6 other compounds. In general, a higher number of metabolites were identified in the ME extract than in the DCM extract (Figure 1). Therefore, 19 phenolic acids and their derivatives were detected in the ME extract, while 10 were identified in the DCM extract.

Various caffeoylquinic acid derivatives were identified as the main phenolic acids in ME. Among all the identified phenolic compounds, the presence of free acids (compounds **2**, **7**, and **18**) was confirmed only in ME, while the rest were hexosides, quinic acid derivatives, and one shikimic acid derivative (compound **12**).

Three proanthocyanidins (compounds **22**–**24**) were identified in ME, while the presence of proanthocyanidins in DCM was not confirmed by UHPLC–MS^4^ Orbitrap metabolic fingerprinting. These three proanthocyanidins had previously been detected in some ferns [13,14,15], but not in *Asplenium* species. Additionally, quercetrin (**49**) was the only flavonoid aglycone identified in the ME extract and was absent in the DCM extract (Table 1, Figure 1).

Of the 24 identified derivatives of flavonoid glycosides, only one was not detected in the *A. ceterach* ME extract (compound **28**), which is specific to the *Polypodium vulgare* L. rhizome water extract [16]. In general, the highest numbers of quercetin and kaempferol derivatives were detected, which gave intense MS^2^ or MS^3^ peaks at 301 and 285 *m*/*z*, respectively. The most common glycosidic group for these derivatives was hexosyl, which gives a neutral loss of 162 Da; hexuronyl (176 Da), rhamnosyl (146 Da), and pentosyl (132 Da) derivatives were also present. Several of these derivatives contained, in addition to the sugar unit, an acyl residue (caffeoyl or methyl-caffeoyl (feruloyl)). The presence of most derivatives of flavonoid glycosides was confirmed by the available literature (Table 1), while the tentative structures of some metabolites were proposed based on their HRMS and MS^4^ data. Among the other metabolites, some non-specific compounds for ferns were found, such as phloretin 3′,5′-di-*C*-hexoside (compound **53**), whose fragmentation pattern is consistent with the available literature [17]. This group of compounds also includes two lignan derivatives (compounds **54** and **55**), which in the first stage of fragmentation (MS^2^) lose the hexose unit (180 Da), indicating that the hexose is attached at the 4′ position of the corresponding lignan. Further fragmentation of these compounds results in peaks corresponding to the loss of a methyl group (15 Da).

The variations in the phytochemical profiles of the two *A. ceterach* extracts confirm the notable chemical diversity of specialized metabolites and can be attributed to differences in the polarity of the extraction solvents. In particular, the ME extract was qualitatively much richer than the DCM extract and was characterized by the presence of polar groups of bioactive compounds, such as phenolic acids, flavonoids, and proanthocyanidins (Table 1, Figure 1). Thus, the results of the current analysis highlight that solvent polarity significantly affects the abundance of compounds found in plant extracts, which is consistent with previous research on other fern species [18].

**Table 1:** HRMS and MS^4^ data for metabolites identified in dichloromethane (DCM) and methanol (ME) extracts of *Asplenium ceterach*.

No	Compound Name	*t*_R_, min	Molecular Formula, [M–H]^−^	Calculated Mass, [M–H]^−^	Exact Mass, [M–H]^−^	Δ ppm	MS^2^ Fragments, (% Base Peak)	MS^3^ Fragments, (% Base Peak)	MS^4^ Fragments, (% Base Peak)	Level of Identification	DCM	ME	Ref.
*Phenolic acids and derivatives*													
1	Dihydroxybenzoyl hexoside	0.91	C_13_H_15_O_9_^−^	315.07216	315.07231	−0.48	109(7), 153(100)	109(100)	81(100)	Level 2a	✚	✚	[19]
2	3,4-Dihydroxybenzoic acid	0.92	C_7_H_5_O_4_^−^	153.01933	153.01970	−2.42	108(13), 109(100), 110(20), 111(26), 123(12), 125(56)	67(100), 81(69)	NA	Level 1	–	✚	[8]
3	Hydroxybenzoyl hexoside	1.33	C_13_H_15_O_8_^−^	299.07724	299.07718	0.21	137(100), 138(7), 153(8), 171(3), 253(5), 261(3)	NA	NA	Level 2b	✚	✚	NA
4	Caffeoyl hexoside 1	1.64	C_15_H_17_O_9_^−^	341.08781	341.08743	1.10	135(5), 179(100)	135(100)	77(6), 79(4), 83(3), 91(16), 107(100), 117(7), 123(4)	Level 2a	✚	✚	[20]
5	Chlorogenic acid hexoside	2.27	C_22_H_27_O_14_^−^	515.14063	515.14078	−0.30	179(5), 191(32), 323(100), 324(14), 341(12), 353(14)	133(6), 161(100)	117(17), 133(100)	Level 2b	–	✚	NA
6	5-*O*-Caffeoylquinic acid	2.89	C_16_H_17_O_9_^−^	353.08781	353.08690	2.56	191(100), 192(5)	85(100), 93(53), 111(34), 127(80), 171(25), 173(53)	55(19), 57(100)	Level 1	✚	✚	[8]
7	Caffeic acid	3.14	C_9_H_7_O_4_^−^	179.03498	179.03521	−1.25	89(4), 119(4), 133(4), 134(6), 135(100)	63(72), 77(52), 91(100), 107(59)	NA	Level 1	–	✚	[8]
8	Coumaroyl hexoside	3.23	C_15_H_17_O_8_^−^	325.09289	325.09259	0.92	119(9), 163(100), 164(9)	119(100)	NA	Level 2a	✚	✚	[21]
9	Feruloyl hexoside	3.68	C_16_H_19_O_9_^−^	355.10346	355.10246	2.80	191(18), 193(100)	134(83), 149(100), 178(60)	134(100)	Level 2b	✚	–	NA
10	5-*O*-Caffeoylquinic acid isomer	3.77	C_16_H_17_O_9_^−^	353.08781	353.08741	1.13	191(100), 192(3)	85(100), 93(59), 111(37), 127(79), 171(24), 173(57)	NA	Level 2a	✚	–	[8]
11	Coumaroylquinic acid 1	3.77	C_16_H_17_O_8_^−^	337.09289	337.09268	0.63	163(4), 173(4), 191(100)	85(100), 93(59), 111(35), 127(77), 171(23), 173(44)	NA	Level 2a	–	✚	[22]
12	4-*O*-Caffeoylshikimic acid	3.96	C_16_H_15_O_8_^−^	335.07724	335.07703	0.63	135(18), 179(100), 180(5)	135(100)	91(31), 106(16), 107(100)	Level 2a	✚	✚	[23]
13	5-*O*-Feruloylquinic acid	4.13	C_17_H_19_O_9_^−^	367.10346	367.10337	0.22	173(4), 191(100), 193(3)	85(100), 93(53), 111(35), 127(94), 171(26), 173(64)	57(100)	Level 2a	✚	✚	[24]
14	Coumaroylquinic acid 2	4.31	C_16_H_17_O_8_^−^	337.09289	337.09303	−0.42	163(3), 179(7), 191(100)	85(100), 109(21), 111(35), 127(84), 171(25), 173(45)	57(100)	Level 2a	–	✚	[22]
15	Me 5-*O*-caffeoylquinate	4.53	C_17_H_19_O_9_^−^	367.10346	367.10355	−0.26	135(45), 136(4), 161(9), 179(100), 180(7), 191(28)	135(100)	68(6), 91(17), 95(12), 107(100), 117(26)	Level 2a	✚	✚	[25]
16	3,5-*O*-Dicaffeoylquinic acid	5.12	C_25_H_23_O_12_^−^	515.11950	515.12044	−1.83	173(3), 179(3), 191(3), 335(4), 353(100), 354(17)	135(9), 173(13), 179(50), 191(100)	93(64), 109(28), 111(34), 127(100), 171(28), 173(65)	Level 2a	–	✚	[26]
17	4,5-*O*-Dicaffeoylquinic acid	5.39	C_25_H_23_O_12_^−^	515.11950	515.11997	−0.91	173(7), 203(14), 255(6), 299(10), 353(100), 354(11)	135(8), 173(100), 179(58), 191(32)	71(16), 93(100), 109(7), 111(49), 137(6), 155(9)	Level 2a	–	✚	[26]
18	Ferulic acid	5.52	C_10_H_9_O_4_^−^	193.05063	193.05093	−1.55	134(64), 161(100), 178(28)	133(100)	78(11), 84(13), 105(100)	Level 1	–	✚	[8]
19	Coumaroyl-caffeoylquinic acid 1	5.55	C_25_H_23_O_11_^−^	499.12459	499.12516	−1.16	335(12), 337(100), 338(14), 353(76), 451(19), 463(46)	119(5), 163(100), 173(77), 191(36)	119(100)	Level 2a	–	✚	[26]
20	Coumaroyl-caffeoylquinic acid 2	5.88	C_25_H_23_O_11_^−^	499.12459	499.12520	−1.23	173(3), 335(4), 337(28), 353(4), 451(100), 452(17)	143(6), 159(10), 161(56), 289(100)	97(4), 101(28), 113(15), 143(11), 159(8), 161(100)	Level 2a	–	✚	[26]
21	Dicoumaroylquinic acid	6.36	C_25_H_23_O_10_^−^	483.12967	483.13020	−1.09	173(10), 319(21), 337(100), 338(9), 435(68), 436(15)	163(19), 173(100), 191(3)	71(15), 93(100), 109(9), 111(58), 127(4), 155(6)	Level 2b	–	✚	NA
*Proanthocyanidins*													
22	B type proanthocyanidin dimer	2.11	C_30_H_25_O_12_^−^	577.13515	577.13423	1.59	287(10), 289(27), 407(50), 425(100), 426(17), 451(23)	273(9), 339(3), 381(6), 407(100)	255(32), 281(88), 283(37), 285(100), 297(39), 389(28)	Level 2a	–	✚	[14]
23	Aesculitannin B	3.93	C_45_H_35_O_18_^−^	863.18289	863.18318	−0.34	411(32), 451(20), 559(12), 573(22), 693(12), 711(100)	407(23), 425(10), 541(26), 559(84), 585(8), 693(100)	407(39), 525(9), 567(100), 570(13), 657(34), 675(9)	Level 2a	–	✚	[13]
24	Cinnamtannin B1	4.11	C_45_H_35_O_17_^−^	847.18797	847.18892	−1.12	411(100), 435(24), 557(21), 649(75), 682(45), 711(26)	149(41), 215(12), 243(14), 257(11), 285(100), 301(14)	125(25), 163(69), 217(43), 241(86), 243(28), 257(100)	Level 2a	–	✚	[15]
*Flavonoid glycosides*													
25	(epi)-Catechin 5-*O*-hexoside	3.18	C_21_H_23_O_11_^−^	451.12459	451.12501	−0.95	289(100), 290(18), 353(20), 354(4), 405(4)	125(5), 179(15), 203(10), 205(35), 245(100), 247(6)	161(18), 187(18), 188(19), 203(100), 227(22), 230(6)	Level 2a	–	✚	[27]
26	Isorhamnetin 3-*O*-(2″-rhamnosyl)-hexoside	3.21	C_28_H_31_O_16_^−^	623.16176	623.16196	−0.33	503(100), 517(12), 521(13), 535(22), 575(21), 605(23)	297(6), 315(26), 359(5), 399(4), 459(100), 485(48)	271(30), 314(9), 315(100), 399(29), 415(19), 441(30)	Level 2b	–	✚	NA
27	Kaempferide 3-*O*-hexoside-7-*O*-rhamnoside	3.72	C_28_H_31_O_15_^−^	607.16684	607.16979	−4.86	341(12), 503(100), 545(24), 547(29), 563(27), 589(28)	189(3), 313(72), 323(14), 341(100), 475(35), 485(5)	165(6), 193(3), 271(3), 313(100), 323(12)	Level 2a	–	✚	[28]
28	(epi)-Catechin 7-*O*-pentoside	3.68	C_20_H_21_O_10_^−^	421.11402	421.11399	0.07	137(39), 138(4), 269(6), 289(100), 290(14), 375(10)	125(18), 137(15), 179(9), 203(8), 205(39), 245(100)	161(28), 187(18), 188(13), 203(100), 217(7), 227(11)	Level 2a	✚	–	[16]
29	Myricetin 3,7-di-*O*-hexoside	3.86	C_27_H_29_O_18_^−^	641.13594	641.13642	−0.75	271(22), 316(100), 317(80), 318(10), 477(13), 623(11)	151(6), 179(14), 270(33), 271(100), 287(42), 288(10)	199(8), 215(9), 227(38), 229(5), 243(100), 271(11)	Level 2a	–	✚	[29]
30	Quercetin 3-*O*-(6″-hexosyl)-hexoside	4.22	C_27_H_29_O_17_^−^	625.14102	625.14145	−0.68	271(11), 300(77), 301(100), 302(14), 343(14), 463(3)	151(75), 179(100), 256(11), 257(11), 272(14), 273(18)	151(100)	Level 2a	✚	✚	[30]
31	Quercetin 3-*O*-(6″-hexosyl)-hexoside-7-*O*-(6″-feruloyl)-hexoside	4.31	C_43_H_47_O_25_^−^	963.24119	963.24174	−0.57	625(100), 626(9)	255(5), 271(8), 300(44), 301(100), 343(10)	151(74), 179(100), 193(5), 229(5), 257(9), 273(18)	Level 2b	–	✚	NA
32	Kaempferol 3-*O*-(6″-hexosyl)-hexoside	4.62	C_27_H_29_O_16_^−^	609.14611	609.14575	0.59	257(3), 284(3), 285(100)	151(23), 197(19), 229(49), 241(35), 257(100), 267(43)	163(45), 189(10), 211(9), 213(23), 229(100), 239(33)	Level 2a	✚	✚	[30]
33	Quercetin 3-*O*-glucoside	4.74	C_21_H_19_O_12_^−^	463.08820	463.08875	−1.19	300(20), 301(100), 302(10)	151(79), 179(100), 257(11), 271(10), 272(10), 273(15)	151(100)	Level 1	–	✚	[31]
34	Quercetin 3-*O*-(6″-pentosyl)-hexoside	4.76	C_26_H_27_O_16_^−^	595.13046	595.13098	−0.87	271(10), 300(56), 301(100), 302(12), 343(9), 463(5)	151(84), 179(100), 256(14), 257(12), 272(23), 273(18)	151(100)	Level 2a	–	✚	[14]
35	Quercetin 3-*O*-hexuronide	4.68	C_21_H_17_O_13_^−^	477.06746	477.06756	−0.20	301(100), 302(11)	1051(81), 179(100), 273(9)	151(100)	Level 2a	✚	✚	[30]
36	Kaempferol 3-*O*-glucoside	4.98	C_21_H_19_O_11_^−^	447.09329	447.09380	−1.15	255(14), 284(100), 285(63)	227(12), 255(100), 256(17)	167(7), 211(74), 227(100)	Level 1	–	✚	[32]
37	Quercetin 3-*O*-(caffeoyl-hexosyl)-hexoside	5.02	C_36_H_35_O_20_^−^	787.17272	787.17291	−0.25	301(3), 383(4), 485(3), 625(100), 626(24)	255(3), 271(5), 300(22), 301(100), 343(7)	151(74), 179(100), 193(6), 229(5), 257(10), 273(17)	Level 2b	–	✚	NA
38	Kaempferol 3-*O*-hexuronide	5.10	C_21_H_17_O_12_^−^	461.07255	461.07285	−0.65	285(100), 286(9)	197(22), 229(55), 239(21), 241(37), 257(100), 267(44)	163(62), 185(11), 187(11), 213(23), 229(100), 239(30)	Level 2b	✚	✚	NA
39	Kaempferol 3-*O*-(6″-pentosyl)-hexoside	5.13	C_26_H_27_O_15_^−^	579.13554	579.13662	−1.86	257(3), 285(100), 286(8)	151(21), 229(51), 241(36), 256(20), 257(100), 267(43)	163(55), 185(12), 187(12), 213(22), 229(100), 239(35)	Level 2a	✚	✚	[33]
40	Quercetin 3-*O*-(6″-caffeoyl)-hexoside-7-*O*-hexoside	5.22	C_36_H_35_O_20_^−^	787.17272	787.17300	−0.36	625(100), 626(16)	255(3), 271(7), 300(26), 301(100), 343(10)	151(79), 179(100), 193(9), 257(15), 273(13), 283(4)	Level 2b	–	✚	NA
41	Kaempferol 3-O-(caffeoyl-hexosyl)-hexoside	5.36	C_36_H_35_O_19_^−^	771.17780	771.17768	0.16	383(7), 485(26), 486(6), 609(100)	255(7), 257(6), 284(27), 285(100), 327(13), 447(3)	151(30), 229(56), 239(21), 241(39), 257(100), 267(42)	Level 2b	–	✚	NA
42	Kaempferol 3-*O*-(2″-caffeoyl)-hexoside-7-*O*-rhamnoside 1	5.73	C_36_H_35_O_18_^−^	755.18289	755.18331	−0.56	285(14), 469(3), 591(9), 609(100), 610(23)	255(8), 257(6), 284(27), 285(100), 327(13), 447(4)	151(26), 229(54), 239(24), 241(38), 257(100), 267(33)	Level 2a	–	✚	[34]
43	Quercetin 3-*O*-(6″-caffeoyl)-hexoside	5.75	C_30_H_25_O_15_^−^	625.11937	625.12253	−5.06	301(100), 302(10), 463(19), 464(4)	151(87), 179(100), 257(14), 273(18)	151(100)	Level 2a	–	✚	[35]
44	3-Me-Kaempferol 7-*O*-hexoside	5.75	C_22_H_21_O_11_^−^	461.10894	461.10857	0.78	283(68), 284(18), 298(66), 446(100)	255(3), 283(100)	255(100)	Level 2a	–	✚	[36]
45	Kaempferol 3-*O*-(2″-caffeoyl)-hexoside-7-*O*-rhamnoside 2	5.93	C_36_H_35_O_18_^−^	755.18289	755.18331	−0.55	285(8), 591(7), 609(100), 610(16)	257(3), 285(100)	151(20), 213(31), 229(53), 241(38), 257(100), 267(46)	Level 2a	–	✚	[34]
46	Kaempferol 3-*O*-(6″-caffeoyl)-hexoside	6.11	C_30_H_25_O_14_^−^	609.12498	609.12526	−0.46	285(100), 286(8), 323(4)	151(100), 229(20), 241(34), 257(62)	83(10), 107(100)	Level 2b	–	✚	NA
47	Luteolin 7-*O*-(2″-caffeoyl)-hexuronide	6.23	C_30_H_23_O_15_^−^	623.10424	623.10485	−0.97	245(5), 285(3), 337(100), 338(10), 443(4)	161(100), 179(4), 203(6), 219(8), 245(13), 277(4)	133(100)	Level 2b	–	✚	NA
48	Apigenin 7-*O*-(2″-caffeoyl)-hexuronide	6.67	C_30_H_23_O_14_^−^	607.10933	607.10998	−1.07	285(40), 286(9), 321(46), 381(11), 442(29), 443(100)	283(4), 284(19), 285(100), 325(3), 371(5), 425(3)	199(23), 213(22), 229(45), 241(38), 257(100), 267(34)	Level 2b	–	✚	NA
*Flavonoid aglycones*													
49	Quercetin	4.68	C_15_H_9_O_7_^−^	301.03538	301.03336	6.68	151(84), 179(100), 257(11), 273(15)	107(2), 151(100)	NA	Level 1	–	✚	[37]
*Other metabolites*													
50	Citric acid	0.78	C_6_H_7_O_7_^−^	191.01973	191.01970	0.12	111(100), 129(3), 173(13)	67(100)	NA	Level 2a	✚	✚	[38]
51	2-(3,4-Dihydroxyphenyl)ethyl hexoside	1.30	C_14_H_19_O_8_^−^	315.10854	315.10862	−0.26	151(11), 153(100), 154(7), 225(5)	123(100)	77(9), 81(25), 93(10), 95(100)	Level 2a	–	✚	[39]
52	Gibberellic acid	4.69	C_19_H_21_O_6_^−^	345.13436	345.13436	0.01	143(24), 239(100), 283(27), 301(30), 327(20), 329(45)	71(16), 83(11), 143(100), 195(11), 197(47), 221(25)	NA	Level 2a	✚	–	[40]
53	Phloretin 3′,5′-di-*C*-hexoside	4.87	C_27_H_33_O_15_^−^	597.18249	597.18276	−0.44	301(41), 357(85), 387(57), 417(20), 477(100), 553(17)	357(100), 369(3), 387(34), 459(7)	167(11), 189(4), 209(100), 229(5), 251(4), 251(15)	Level 2b	–	✚	NA
54	Hydroxypinoresinol 4′-*O*-hexoside	5.41	C_26_H_31_O_12_^−^	535.18210	535.18293	−1.55	355(100), 356(25), 489(4)	323(4), 340(100)	253(4), 267(100), 296(4), 312(4)	Level 2b	–	✚	NA
55	Medioresinol 4′-*O*-hexoside	6.08	C_27_H_33_O_12_^−^	549.19775	549.20035	−4.74	354(15), 369(100), 370(17), 487(19), 505(12), 531(12)	337(7), 353(9), 354(100)	339(100)	Level 2b	✚	✚	NA

Identification confidence levels are assigned according to the Schymanski scale: Level 1—confirmed structure via reference standard; Level 2—probable structure (a: library match, b: diagnostic evidence); *t*_R_—retention time (min); Δ ppm—mean mass accuracy; DCM—dichloromethane extract; ME—methanol extract; ✚ stands for detected and – stands for not detected compound; NA—not available.

### 2.2. High-Performance Thin-Layer Chromatography (HPTLC) Phytochemical and Bioactivity Profiles of A. ceterach Extracts

#### 2.2.1. Phenolic/Terpenoid Fingerprints of *A. ceterach* Extracts

Polyphenols are effective ROS scavengers, and their accumulation was established in several resurrection plants [41,42,43]. Accordingly, a high number of these compounds were identified in different extracts of *A. ceterach* sporophytes grown under normal conditions, suggesting their importance for desiccation and oxidative stress tolerance [44].

**Figure 2:**
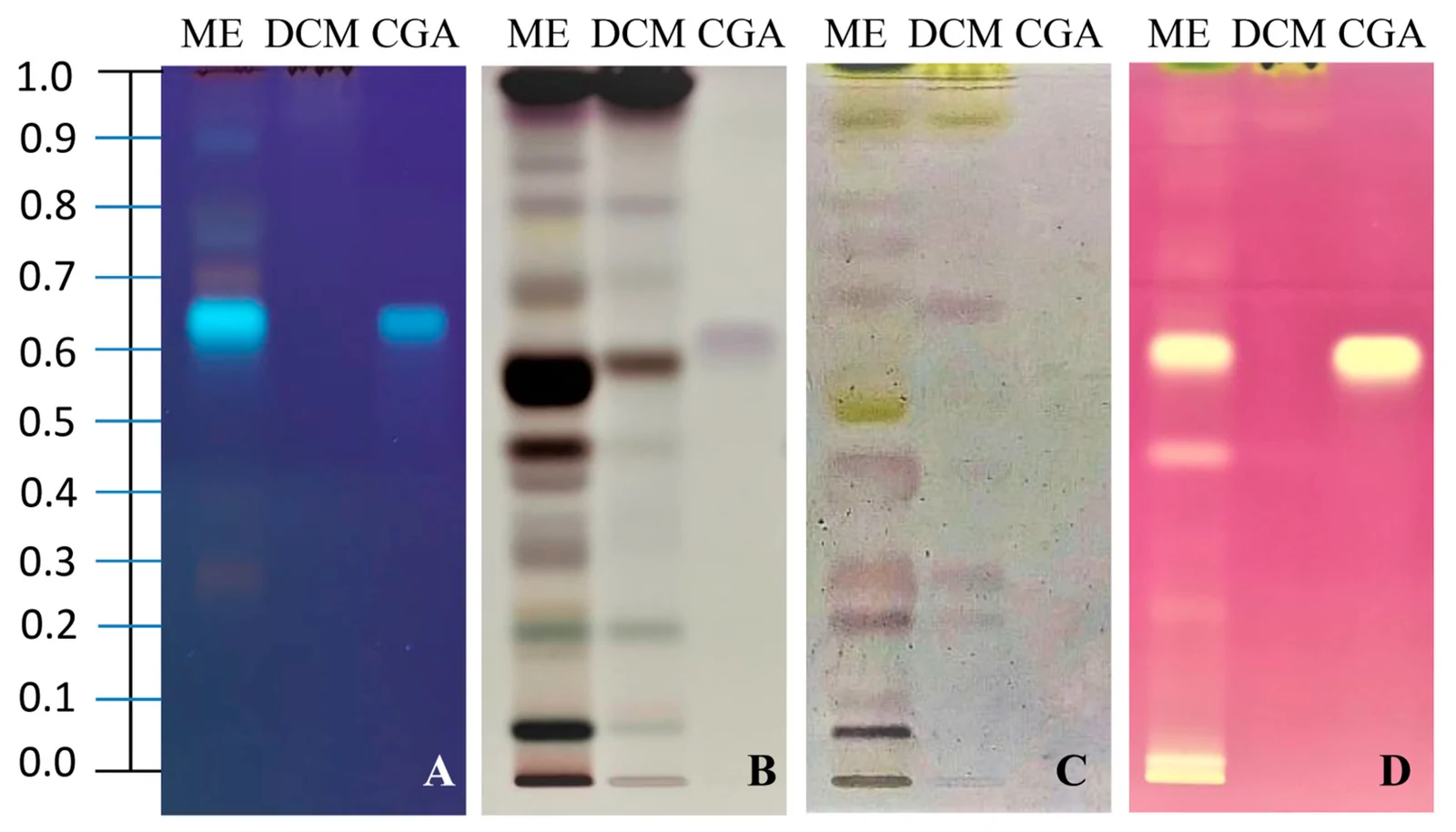
HPTLC profiles of ME (methanol) and DCM (dichloromethane) extracts of *A. ceterach* visualized under: (**A**) UV 366 nm; (**B**) visible light after *p*-anisaldehyde derivatization; (**C**) visible light after α-amylase derivatization; (**D**) visible light after DPPH derivatization. Abbreviations: CGA—chlorogenic acid.

HPTLC and HPTLC-bioassays were used to obtain the phytochemical and bioactivity profiles of the ME and DCM extracts of *A. ceterach* sporophytes. Screening at 366 nm leveraged the native fluorescence of phenolic cores, yielding distinct blue, yellow, orange, and green zones [45] (Figure 2A). Under UV 366 nm, the ME extract exhibited a remarkably richer phenolic profile than the highly depleted DCM extract. A prominent blue zone at *R*_F_ 0.64 in ME was identified as chlorogenic acid (5-*O*-caffeoylquinic acid, CGA), indicating its high abundance in ME, as confirmed in the literature [46,47,48,49]. Additionally, lower-intensity phenolic zones at *R*_F_ 0.27, 0.70, 0.76, 0.79, and 0.90 were detected in ME. This HPTLC phenolic fingerprint strongly correlates with the UHPLC data, which revealed numerous caffeoylquinic acids in ME that were completely absent in DCM; the intense blue zone likely arises from single or co-eluting caffeoylquinic derivatives, including CGA. Conversely, the terpenoid profile (Figure 2B) was far more complex for both samples, with diterpenes emerging as the most abundant terpenoid class in this plant [50].

Within this fraction, a series of intense brown zones was identified as diterpenic compounds, led by a prominent band at *R*_F_ 0.58 unique to ME. ME also displayed distinct diterpene bands at *R*_F_ 0.08 and 0.47. Notably, the most prominent diterpenoid zone was detected near the solvent front at *R*_F_ 0.98 in both samples, appearing slightly more intense in the DCM extract. Additionally, both samples contained a distinct constituent at *R*_F_ 0.22, visualized as a green zone, strongly suggesting the presence of a triterpenoid derivative or sterol glycoside [51]. The DCM extract exhibited a structurally related brown zone at *R*_F_ 0.60. The remaining gray-purple zones across the chromatogram of both extracts (0.32, 0.42, 0.69, 0.82, 0.85) most likely correspond to mono- and/or triterpenoids, further highlighting superior terpenoid complexity.

#### 2.2.2. HPTLC-α-Amylase Assay

The results demonstrated that the ME extract possesses relatively higher *α*-amylase inhibitory activity compared to the DCM extract. Comparative evaluation of the HPTLC phytochemical and bioactivity profiles (Figure 2A–C) indicated that the observed inhibition may arise from chemically diverse constituents. Both extracts exhibited common bioactive zones at *R*_F_ values of 0.22 and 0.29; however, these zones were noticeably more intense in the ME extract. The ME extract displayed additional prominent active zones at *R*_F_ 0.47, 0.69 and 0.76. Conversely, a distinct active zone at *R*_F_ 0.66 was uniquely observed in the DCM extract. Some of these inhibitory zones, particularly one at *R*_F_ 0.47, overlapped with the *p*-anisaldehyde-positive zone, suggesting a possible contribution of terpenoid-like constituents. Conversely, the zone at *R*_F_ 0.69, visualized in both *p*-anisaldehyde and NEU/PEG, and the active zone at *R*_F_ 0.76, corresponding to a region of the phenolic fingerprint, indicate that phenolic constituents may also contribute to the observed inhibition. Indeed, terpenoids, including di- and triterpenes, as well as saponins, have been reported as potential inhibitors of carbohydrate-hydrolyzing enzymes such as *α*-amylase and *α*-glucosidase, contributing to their antidiabetic potential, supporting our findings [52]. Polyphenols may also contribute to the observed activity, as fern-derived flavonoids have previously demonstrated α-amylase inhibitory effects, potentially mediated by interactions with the enzyme, including hydrogen bonding [53]. Overall, the observed activity likely results from the combined contribution of chemically diverse constituents.

Notably, despite being the most prominent region in the ME phytochemical fingerprint, the CGA/caffeoylquinic acid region (*R*_F_ 0.64) did not exhibit pronounced α-amylase inhibitory activity in our HTPLC-α-amylase analysis. This observation is consistent with studies describing CGA as a relatively weak α-amylase inhibitor [54], although higher inhibitory activity has been reported under different experimental conditions [55]. Nevertheless, other CGA derivatives identified in ME may contribute to the observed inhibition, as several caffeoylquinic acid derivatives have been reported as α-amylase inhibitors [54,55].

#### 2.2.3. HPTLC-DPPH Assay

Considering the HPTLC-DPPH bioautogram, the ME extract demonstrated a significantly higher antioxidant potential than DCM (Figure 2D). The prominent DPPH-active zone corresponding to CGA indicated caffeoylquinic acids as major contributors to the antioxidant activity of the ME extract, whereas other phenolic zones (*R*_F_ > 0.70) showed comparatively weak activity [56]. Nevertheless, non-phenolic constituents also contributed to the antioxidant profile, as indicated by the moderate activity of the *p*-anisaldehyde-positive zone near the solvent front (*R*_F_ 0.98) in both extracts and the prominent active zone at *R*_F_ 0.47 in ME.

The HPTLC-DPPH bioautogram identified caffeoylquinic acids as the main contributors to the antioxidant activity, whereas phenolic zones at *R*_F_ > 0.70 showed negligible activity. A common terpenoid zone at *R*_F_ 0.98 exhibited moderate activity in both extracts. In ME, the second most potent antioxidant contributor was the terpenoid zone at *R*_F_ 0.47, supplemented by highly polar, non-phenolic/non-terpenoid constituents remaining at the application baseline (*R*_F_ 0.00 and 0.02). Additional antioxidant activity was observed for highly polar constituents remaining near the application zone (*R*_F_ 0.00–0.02), which may include highly polar phenolic compounds that were strongly retained on the normal-phase silica. Thus, comparative evaluation of the ME’s HPTLC fingerprints indicates that the antioxidant activity of the extracts results from contributions of chemically diverse constituents, with CGA/caffeoylquinic acids representing the most prominent contributors in the ME extract.

The antioxidant potential of various *A. ceterach* extracts has been extensively studied, including their DPPH radical scavenging capacity (for details see [7]). Generally, the capacity to scavenge radicals was concentration-dependent and increased with higher concentrations of the extract. Moreover, the antioxidant activity of *A. ceterach* was strongly correlated with the type of extract, with greater radical absorbance capacity observed for polar solvents compared to nonpolar ones [57]. Previous studies showed that the methanol extract of *A. ruta-muraria* exhibited significantly high DPPH antioxidant potential, presumably due to flavonoids, which were the dominant phenolic compounds [8]. Jarial et al. [58] reported that the maximum yield of extracted flavonoids from *Asplenium nidus* had remarkable DPPH radical-scavenging activity. In addition, the free radical-scavenging activity test showed that the ethyl acetate extract of *Asplenium trichomanes* exhibited higher antioxidant activity than extracts in less polar solvents. Furthermore, a significant correlation was found between levels of phenolics in ethyl acetate extracts of *A. trichomanes* and its antioxidant potential [59].

### 2.3. EPR Measurement of Scavenging Activity Towards DPPH and Hydroxyl Radicals

**Figure 3:**
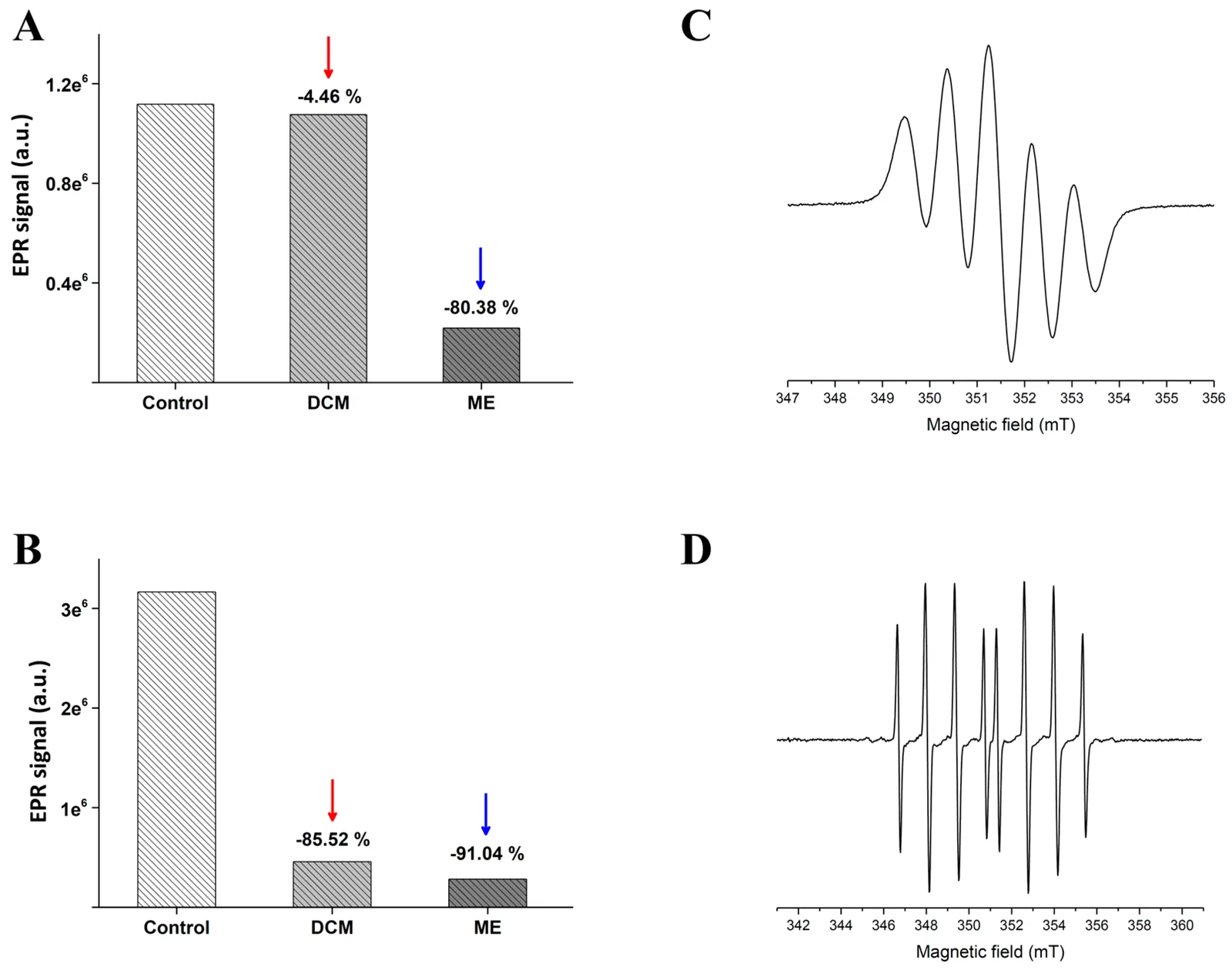
Radical scavenging activity of *A. ceterach* extracts against (**A**) DPPH and (**B**) hydroxyl radicals (•OH). Activity is expressed as the reduction in peak intensity (%) obtained 2 min after the addition of DPPH and/or •OH generation in the Fenton reaction system containing the spin trap DEPMPO. Representative EPR spectra of DPPH (**C**) and DEPMPO/OH spin adduct (**D**). Abbreviations: DCM—diclormethane extract of *A. ceterach*; ME—methanol extract of *A. ceterach*.

The in vitro DPPH radical scavenging activity of the extracts was also assessed using EPR spectroscopy, and results are presented in Figure 3A,C, where a higher percentage reduction in signal intensity indicates greater radical scavenging activity against DPPH.

Interestingly, higher anti-DPPH activity was recorded for the ME extract (80.38%) compared to the DCM extract (4.46%), which aligns with literature data showing that phenolics are often extracted in higher amounts with more polar solvents [60,61,62,63]. This finding is also consistent with phytochemical fingerprint reports, which showed that the ME extract is qualitatively richer in polyphenolics than the DCM samples; that is, significantly more redox-active components were found in the ME extract (Table 1, Figure 1). Considering the profile of redox-active components obtained by OrbiTrap analysis and the presented EPR results, there is strong evidence that phenolic acids and flavonoid glycosides, together with procyanidins, are responsible for the antioxidative capacity of *A. ceterach* extracts. To determine how the selected extracts are capable of removing biologically relevant short-lived radical species, such as ^•^OH, the Fenton reaction containing the spin trap DEPMPO was used. EPR spectroscopy enables direct and specific detection of short-lived radical species, offering a reliable approach for assessing ^•^OH radical scavenging activity in biological samples [64]. As shown in Figure 3B,D, fern extracts caused a decrease in the EPR signal intensity of DEPMPO/OH spin-adducts. Both ME and DCM extracts of *A. ceterach* demonstrated a remarkable scavenging effect against ^•^OH radicals (91.04% and 85.52%, respectively). According to Kukavica and co-workers [64], the ethanol extract of *A. ceterach* fronds also exhibited significant free radical scavenging and reducing abilities, as confirmed by various methods.

### 2.4. Antimicrobial and Antibiofilm Activity of A. ceterach Extracts

The antibacterial and antifungal activity of two extracts of *A. ceterach* sporophyte was comparatively tested against six bacterial and six fungal pathogens using the microdilution method. The results are presented as MIC and MBC/MFC in Table 2. Besides the standard tested bacterial strains, some clinical and food isolates were used as well.

Most food-borne illnesses are caused by *S. aureus*, *B. cereus*, *L. monocytogenes*, *S. Typhimurium* and *E. coli*, while *S. aureus*, *P. aeruginosa*, and *E. coli* give rise to some hospital-acquired infections. According to the results of the antibacterial test, both plant extracts showed noticeable activity against tested strains, although in variable degree. Minimal inhibitory concentration (MIC) for both extracts ranged from 0.25 to 1.50 mg mL^−1^, and minimal bactericidal concentration (MBC) was 0.75–2.00 mg mL^−1^. Although both extracts possessed lower antimicrobial activity compared to the standard antimicrobial agent streptomycin, the obtained results indicate that ME extract showed stronger antimicrobial activity than DCM. The Gram (−) bacterium *E.coli* and the Gram (+) bacterium *B. cereus* were the most sensitive bacterial species, since they were inhibited by all tested extracts (MIC values ranged from 0.25 to 0.75 mg mL^−1^). Several studies have shown that fern extracts have an antibacterial effect on both Gram (+) and Gram (−) bacteria; hence, the effect was more pronounced on Gram (+) bacteria. According to Petkov et al. [37], the methanol extract of *A. ceterach* showed activity against *P. aeruginosa,* while extracts of *A. trichomanes* and *A. scolopendrium* had no effect. On the other hand, extracts of all three *Asplenium* species exhibited an antibacterial effect on the Gram (+) bacterium *B. cereus*. Similarly, methanol extract of *Asplenium nidus* [65], *A. adiantum–nigrum,* and *A. ruta muraria* [8] exhibited significant activity against different Gram (−) bacteria. In addition, ME extract of *A. incisum* decreased the proliferation of the Gram (−) bacterium *Porphyromonas gingivalis* with sustained antibacterial activity for up to three days [66].

As shown in Table 2, ME and DCM extracts of *A. ceterach* possessed similar antifungal and anti-candidal activities, with MIC values ranging between 0.25 mg mL^−1^ and 1.50 mg mL^−1^, whereas fungicidal activities were within the 0.50–2.00 mg mL^−1^ range. The examined extracts displayed slightly weaker antifungal activity compared to ketoconazole (MIC 0.05–0.25 mg mL^−1^ and MFC 0.10–0.50 mg mL^−1^), which was used as a positive control. The most sensitive of all tested fungal species was the yeast *C. krusei,* with MIC ranging from 0.25 to 1.00 mg mL^−1^. The antifungal screening of *A. adiantum-nigrum* L. and *A. ruta-muraria* L. revealed that the frond ME extracts of both species were more effective against *P. funiculosum* than the commercial fungicides ketoconazole and bifonazole [8]. According to Chan and coworkers [67], the ME extracts of *A. nidus* showed fungicidal activity against the yeast species *Issatchenkia orientalis* with MFC values of 0.63 mg mL^−1^. In addition, it was recently shown that the ME extract of *A. dalhousiae* fronds displayed a pronounced inhibitory effect against *Alternaria* sp., while its antifungal activity against fungal strains such as *Carvularia*, *Fusarium*, *Rhizopus*, and *Aspergillus* was significantly lower [68]. Results regarding the antimicrobial activity of *A. ceterach* extracts used at concentrations equivalent to those applied with commercial antibiotics (<100 mg mL^−1^) clearly indicated a great bactericidal/fungicidal potential of fern extracts at a level comparable to antibiotics [69] and further support their use in traditional medicine against diseases caused by different microorganisms.

The tested extracts of *A. ceterach* fronds have shown significant capability to prevent *C. albicans* biofilm establishment (Table 3).

The highest levels of biofilm inhibition were observed for the extracts applied in concentrations equal to MIC and 0.5 MIC, with no significant difference between the two extracts examined (inhibition range 72–74%). The lowest concentration tested (0.25 MIC) resulted in the least inhibition of biofilm formation, with nearly 2.5 times higher inhibitory activity of DCM (48% inhibition) compared to ME extract (20% inhibition). Similar results were obtained for the ME extracts of *A. ruta-muraria* and *A. adianthum-nigrum* when tested for their potential to inhibit *C. albicans* biofilm formation and prevent initial cell attachment. Specifically, the ME frond of these two species exhibited significant antibiofilm activity even when applied at the sublethal concentrations of 0.5 MIC (inhibition ranging from 32% to 50%) and 0.25 MIC (8–37% inhibition) [8].

The excessive use of antibiotics significantly contributes to the development of bacterial resistance. In addition, biofilm formation is a bacterial resistance mechanism that acts as a physical barrier, metabolically limiting the effectiveness of antibiotic treatment [70]. Various plant-derived bioactive compounds have demonstrated potential antimicrobial and antibiofilm activity through mechanisms such as cell membrane disruption, inhibition of bacterial motility, proliferation, and adhesion, and interruption of quorum sensing (QS), which facilitates bacterial cell–cell communication [71]. The literature confirms that fern extracts are effective antibiofilm agents, showing biofilm-inhibitory activity in various model bacteria, including *E. coli*, *S. aureus*, *P. aeruginosa*, *K. pneumoniae*, *Acinetobacter baumannii*, *S. flexneri* [72,73], and *S. epidermidis* [74]. The percentage reduction in biofilm formation varied depending on the fern species and the polarity of the solvents used and was effective even at concentrations below 10 mg mL^−1^ in *S. aureus* and *E. coli* [73]. These findings indicate that fern extracts may play an important role in developing novel antibacterial agents to address the growing challenge of antibiotic resistance. There is also an urgent need to standardize methods and cut-off points for describing antimicrobial activities, as some authors report activities of extracts at concentrations above 10 mg mL^−1^ while others, including the current group of authors, consider only MIC values below 100 µg mL^−1^ (0.1 mg mL^−1^ for extracts) and 10 µg mL^−1^ (0.01 mg mL^−1^ for compounds) to be worthy of the label “active”.

### 2.5. Cytotoxic Activity of A. ceterach Extracts

**Figure 4:**
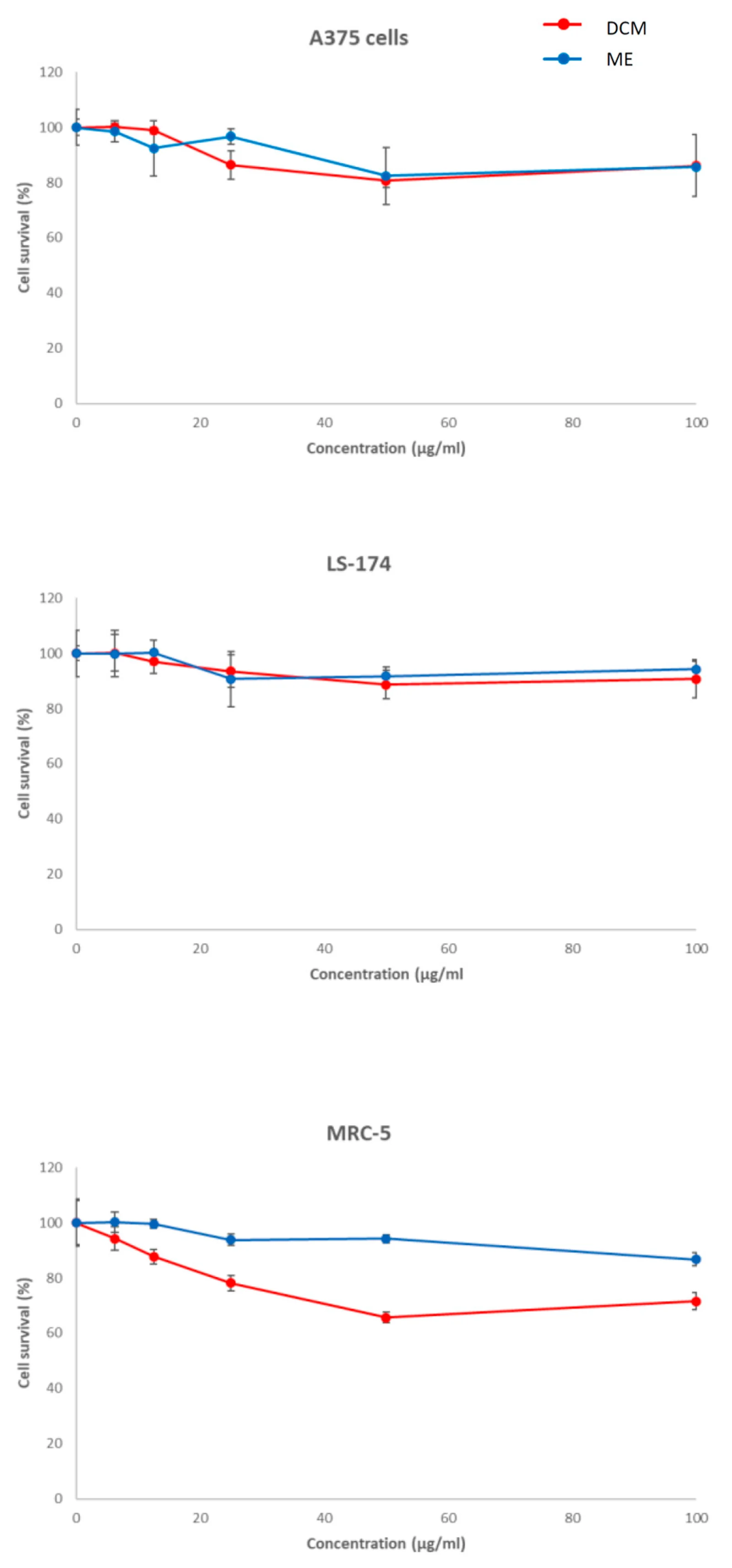
Cytotoxic activity of *A. ceterach* extracts. Percentage of A375, LS-174, and MRC-5 cell growth inhibition (cell viability) was determined by MTT assay after 72 h of continuous treatment with ME (blue line) and DCM (red line) extracts. The values represent results of two independent measurements ± SE. Abbreviations: DCM—dichloromethane extract of *A. ceterach*; ME—methanol extract of *A. ceterach*.

The cytotoxic activity of ME and DCM extracts from *A. ceterach* sporophytes on human melanoma cells (A375), human colon cancer cell lines (LS-174), and human fibroblast cells (MRC-5) was assessed using the MTT assay. The results are shown in Figure 4. Both extracts, after 72 h of continuous incubation, exhibited no cytotoxic activity on any of the tested cell lines at concentrations up to 100 μg mL^−1^. Furthermore, the extracts did not reduce cell survival below 60% at the highest concentration applied, clearly indicating the absence of cytotoxic potential in the tested cell lines. Cisplatin was used as a standard cytotoxic agent and exhibited IC_50_ values of 3.91 µg mL^−1^ in A375 cells, 20.22 µg mL^−1^ in LS-174 cells, and 6.45 µg mL^−1^ in MRC-5 cells.

The morphological characteristics of A375, LS-174, and MRC-5 cells following treatment with the extracts were analyzed using inverted microscopy. After 72 h of continuous treatment, the cell number was not reduced, and the cells retained their normal morphology, indicating an absence of cytotoxic activity within the investigated concentration range of fern extracts. These findings are consistent with the results obtained from the MTT assay (Supplementary Figure S1). Similarly, Farrás and coworkers [75] reported that ME extract of both *A. ceterach* and *A. trichomanes* fronds did not show cytotoxic effects on several human cancer cell lines (HeLa, HepG2, MCF-7, and A549) when applied at concentrations ranging from 0.01 to 1 mg mL^−1^. The methanolic extract of *A. adiantum-nigrum* L. fronds also did not exhibit cytotoxicity in non-tumor (3T3 and HaCaT) or tumor (HeLa, HepG2, and A549) cell lines [76]. In contrast, Petkov et al. [37] showed that the 70% aqueous methanol extract of *A. ceterach* exhibited the strongest cytotoxic activity towards HeLa cells compared to extracts of *A. trichomanes* and *A. scolopendrium*. Based on data on the chemical composition, Petkov et al. [37] suggested that the presence of phenolic compounds, primarily kaempferol and its derivatives, as well as tannins and ω-3 polyunsaturated fatty acids (PUFAs), contributed to the observed cytotoxic activity of the *A. ceterach* extract, although other compounds could also contribute. Similarly, Fatima et al. [77] showed that aqueous extract of *A. ceterach* has anticancer activity against two prostate cancer cell lines (PC3 and LNCaP). This extract was rich in phenols (~465 mg per 100 g) and flavonoids (~66 mg/100 g).

Additionally, ethanolic extracts of *A. aethiopicum* exhibited dose- and time-dependent cytotoxic activity against human A549 lung adenocarcinoma and African Green Monkey Kidney (VERO) cells, with IC_50_ values of 381.68 mg mL^−1^ and 714.28 mg mL^−1^, respectively [78]. The authors highlighted the presence of various bioactive compounds in the ethanolic extract of *A. aethiopicum*, which could contribute to its notable anticancer properties compared to other extracts prepared with solvents of different polarity (ether, acetone, chloroform, and water) used in this study. It is noteworthy that the biological activity of the plant extracts is influenced not only by major but also by minor components, as well as their possible synergistic and antagonistic effects [79,80,81]. This is probably the reason for the observed differences in the cytotoxic activity of various *A. ceterach* extracts.

## 3. Materials and Methods

### 3.1. Plant Material and Extracts Preparation

Mature sporophytes of *Asplenium ceterach* L. were collected in East Serbia near the Monastery Gornjak (44°15′52.66″ N 21°32′040.21″ E) and grown in a greenhouse of the Institute for Biological Research “Siniša Stanković”—National Institute of the Republic of Serbia, University of Belgrade (IBISS), at 25 ± 2 °C and a relative humidity of 60–90%. The species was authenticated by the authors, and the corresponding voucher specimens have been deposited at the Department of Plant Physiology, IBISS. Aerial plant parts (fronds) were ground and extracted with methanol (ME) or dichloromethane (DCM) (*v*:*w* = 10:1) (Fisher Scientific, Waltham, MA, USA), using an ultrasonic bath (RK100, Bandelin, Berlin, Germany) at room temperature. After centrifugation for 20 min at 13,400 rpm, the supernatants were collected and filtered through 15 mm RC filters with a 0.22 µm pore size (Econofilter, Agilent Technologies, Santa Clara, CA, USA) and kept in tinted vials at 4 °C until use. All extractions were performed in biological triplicate. For antimicrobial analysis, the extracts were further evaporated to dryness at room temperature, using a vacuum evaporator (Concentrator 5301, Eppendorf, Germany). Each dry extract was dissolved in 30% ethanol to a concentration of 20 mg mL^−1^ and stored in the dark at 4 °C until use.

### 3.2. UHPLC-LTQ Orbitrap MS Untargeted Metabolomics Analysis

Samples (fronds from mature sporophytes) were extracted with ME and DCM as described in Section 2.1.

LC/MS analysis was performed using an Accela UHPLC system (Thermo Fisher Scientific, Bremen, Germany) coupled to an LTQ OrbiTrap mass spectrometer equipped with a heated electrospray ionization (HESI) probe (Thermo Fisher Scientific, Bremen, Germany). An LC-MS method for metabolite profiling of two different *A. ceterach* extracts was performed as reported elsewhere [82].

A Syncronis C18 column (100 × 2.1 mm, 1.7 μm particle size) at 40 °C was used for compound separation. The flow rate was set at 0.250 mL/min, and the mobile phase consisted of (A) water + 0.1 % formic acid (MS grade) and (B) acetonitrile + 0.1 % formic acid. The injection volumes were 5 μL, and the linear gradient programs were as follows: 0.0–1.0 min at 5 % (B), 1.0–14.0 min from 5 % to 95 % (B), 14.0–14.1 min from 95 % to 5 % (B), and 5 % (B) for 6 min.

The MS spectra were acquired by full-range acquisition covering 100–1000 *m*/*z*. Resolution was set to 30,000 for full scan analysis. The data-dependent MS/MS events were always performed on the most intense ions detected in the full-scan MS. The ions of interest were isolated in the ion trap with an isolation width of 5 ppm and activated with 35 % collision energy.

R Studio software (version 2023.09.1, build 494, Posit Software, PBC, Boston, MA, USA) was used for raw untargeted LC-MS data extraction. The “internal” script, based on various R packages, such as the enviPick and xcms R packages [83], included extraction of retention times, exact masses, MS^2–4^ fragments with relative abundances, and prediction of molecular formulas. Furthermore, the filtering of the thus-obtained large data set with the aim of finally retaining only the peaks of interest was performed manually, with emphasis based on the polarity of the compounds and double bond equivalents (DBE). Annotation of the peaks was done through the SciFinder database [84] by searching the mentioned database using keywords, chemical and molecular formulas, and also the study of MS^2–4^ fragmentation.

Classification of the identified compounds (Table 1) was done according to confidence levels following the Schymanski framework [85].

### 3.3. High Performance Thin Layer Chromatography (HPTLC)

Chromatographic separation was performed on HPTLC silica gel 60 F_254_ plates (Merck, Darmstadt, Germany). The extracts, together with a methanolic standard solution of chlorogenic acid (1 mg mL^−1^; Sigma-Aldrich, Steinheim, Germany), were applied to the plates as 8 mm bands using a semi-automatic sampler (Linomat 5, CAMAG, Muttenz, Switzerland). Chromatographic conditions were performed using an optimized procedure as described by Milutinović et al. [82]. The application volume for all samples was precisely 2 µL. Chromatograms were developed at room temperature in a twin-trough chamber, previously saturated with a mobile phase system of ethyl acetate–formic acid–acetic acid–water (100:11:11:27, *v*/*v*/*v*/*v*) tailored for the efficient resolution of the species’ characteristic phenolic compounds [86]. After development, the HPTLC chromatograms were dried in a stream of warm air for 3 min and subsequently derivatized or detected using different reagents to reveal the presence of bioactive constituents. After detection, the HPTLC chromatograms were documented using a mobile phone camera (Huawei P20 lite) and saved in TIFF format for further analysis and archiving.

#### 3.3.1. HPTLC Phenolic Profiles

For the detection of phenolic compounds, the developed HPTLC plates were visualized under UV light at λ = 366 nm, exploiting the inherent natural fluorescence of the separated phenolic constituents.

#### 3.3.2. HPTLC Terpenoid Profiles

To visualize the terpenoid profiles, the developed HPTLC plates were derivatized with *p*-anisaldehyde reagent following well-established literature procedures [87]. Upon derivatization, the presence of diterpenes was confirmed by the appearance of brown zones, whereas gray and purple zones indicated mono- and triterpenes, respectively.

#### 3.3.3. HPTLC-α-Amylase Assay

Amylase inhibition assays were carried out according to [88], using a starch-iodine complexation test for visualization. According to the literature, differences in zone coloration are directly related to the degree of starch hydrolysis. Intact amylose chains, which require a critical length to form stable helices, produce a characteristic, dark-blue complex with iodine, whereas partially hydrolyzed starch fractions, such as shorter-chain dextrins, form complexes that shift the absorption spectrum, resulting in purple or reddish-brown zones. The inactive zones appeared as white or pale backgrounds, indicating no colored complex formation with the iodine solution.

#### 3.3.4. HPTLC-DPPH Assay

The HPTLC-DPPH assay was performed according to the procedure described by Milutinović et al. [82]. Following chromatographic development, the chromatogram was immersed in a 0.1% (*w*/*v*) solution of DPPH radical in methanol. After incubation in the dark, antioxidant-active compounds appeared as yellow zones against a purple background, corresponding to the reduction of the DPPH radical in the presence of bioactive constituents.

### 3.4. EPR Measurements of Antioxidant Activity

The antioxidant activity was measured by an electron paramagnetic resonance (EPR) spectrometer (Bruker ELEXSYS-II spectrometer, Rheinstetten, Germany) operating in X-band. EPR spectra were analyzed using Xepr software, version 2.6b.84 (Bruker BioSpin GmbH, Rheinstetten, Germany), following the method described by Nakarada et al. [85] and Milutinović et al. [82]. To evaluate the capacity of extracts to remove ^•^OH radicals, a Fenton reaction containing the spin trap DEPMPO was deployed as previously described [82,89]. The reaction system (final volume: 29 μL) was prepared by combining 1 μL of extract solution (previously diluted 1000-fold with water), 25 μL of deionized water, 2 μL of H_2_O_2_ (final concentration 0.35 mM), and 1 μL of DEPMPO spin trap (final concentration 3.5 mM). The reaction was initiated by adding 1 μL of FeSO_4_, yielding a final concentration of 0.15 mM. Immediately after preparation, the mixture was transferred into a gas-permeable Teflon capillary tube, and EPR measurements were performed after 2 min incubation at room temperature (293 K). Spectral acquisition was carried out under the following conditions: center field 350.0 mT, microwave power 10 mW, microwave frequency 9.85 GHz, modulation frequency 100 kHz, and modulation amplitude 0.1 mT. For control measurements, the extract was omitted and replaced with the corresponding volume of solvent. Hydroxyl radical scavenging activity (*AA*) was determined according to the equation:

$$AA=\frac{I_{c}-I_{a}}{I_{C}}100\;\left(\%\right)$$

where *I_c_* represents the double integral value obtained for the control sample, while *I_a_* corresponds to the sample containing the extract. These values were calculated from the recorded EPR spectra using Xepr software.

The potential of extracts to scavenge DPPH radicals was investigated using previously developed methodology [90]. A 1 µL aliquot of the extract (previously diluted 1000-fold with water) was mixed with 29 µL of a freshly prepared 210 µM DPPH solution in ethanol. The reaction mixture was left to incubate for 2 min at room temperature, and then the EPR signal was immediately recorded under the following parameters: center field 350.0 mT, microwave power 10 mW, microwave frequency 9.85 GHz, modulation frequency 100 kHz, and modulation amplitude 0.2 mT. For control measurements, the same volume of pure ethanol was used in place of the extracts.

### 3.5. In Vitro Antimicrobial and Antibiofilm Activity

*A. ceterach* extracts were tested against Gram (+) bacteria: *Bacillus cereus* (food isolate Bc-AC-11), *Staphylococcus aureus* (ATCC 11632) and *Listeria monocytogenes* (NCTC 7973), Gram (−) bacteria *Escherichia coli* (ATCC 25922), *Pseudomonas aeruginosa* (ATCC 27853), and *Salmonella Typhimurium* (ATCC 13311) and fungi: *Aspergillus fumigatus* (ATCC 204305), *Aspergillus niger* (ATCC 6275), *Penicillium funiculosum* (ATCC 36839), *Penicillium verrucosum* var. *cyclopium* (food isolate Pvc–DS–11), *Candida albicans* (ATCC 10231) and *Candida krusei* (clinical isolates K1/16). The bacterial strains were cultured on solid Tryptic Soy agar (TSA), while micromycetes were cultured on solid malt agar (MA) and yeast were sustained on Sabouraud dextrose agar medium (SDA). The cultures were subcultured once a month and stored at 4 °C for further utilization. All the tested microorganisms are deposited at the Mycological Laboratory, Department of Plant Physiology, Institute for Biological Research “Siniša Stanković”—National Institute of the Republic of Serbia, University of Belgrade. The minimum inhibitory and bactericidal/fungicidal concentrations (MICs, MBCs/MFCs) and antibiofilm activity of plant extracts were determined as reported earlier [8].

### 3.6. Cytotoxic Activity

#### 3.6.1. Reagents

3-(4,5-dymethylthiazol-yl)-2,5-diphenyltetrazolium bromide (MTT), dimethyl sulfoxide (DMSO), and sodium dodecyl sulfate (SDS) were obtained from Sigma-Aldrich (Sigma Chemicals Co., St. Louis, MO, USA), as well as all culture media and serum.

#### 3.6.2. Cell Culture

The human melanoma A375 (ATCC, CRL-1619), human colon carcinoma LS-174 (ATCC, CL-188), and human fetal lung fibroblast MRC-5 (ATCC, CCL-171) cell lines were maintained as monolayer cultures in the Roswell Park Memorial Institute (RPMI) 1640 nutrient medium (Sigma Chemicals Co., St. Louis, MO, USA). RPMI 1640 nutrient medium (Gibco, Grand Island, NY, USA) was prepared in sterile ionized water, supplemented with penicillin (100 IU mL^−1^), streptomycin (200 µg mL^−1^), 4-(2-hydroxyethyl)piperazine-1-ethanesulfonic acid (HEPES) (25 mM), L-glutamine (3 mM), and 10% heat-inactivated fetal calf serum (FCS) (pH 7.2). The cells were grown at 37 °C in a humidified atmosphere containing 5% CO_2_.

#### 3.6.3. MTT Assay

The cytotoxicity of the investigated extracts and cisplatin (as a reference compound) was determined using the 3-(4,5-dimethylthiazol-yl)-2,5-diphenyltetrazolium bromide assay as previously described [91]. Briefly, 24 h after seeding the cells into 96-well cell culture plates (Thermo Scientific Nunc™, Rochester, NY, USA), the cells were exposed to the investigated extracts, which were dissolved in DMSO at a concentration of 10 mg mL^−1^ and afterward diluted in culture medium to the desired concentrations. The final DMSO concentration never exceeded 1% (*v*:*v*). After an incubation period of 72 h, 20 μL of MTT solution (5 mg mL^−1^ in phosphate buffer, pH 7.2) was added to each well. Samples were incubated for 4 h at 37 °C in a humidified atmosphere of 5% CO_2_, and then 100 μL of 10% sodium dodecyl sulfate (SDS) was added. Absorbance was recorded after 24 h, on an enzyme-linked immunosorbent assay (ELISA) reader (Thermo Labsystems Multiskan EX, Waltham, MA, USA; 200–240 V) at a wavelength of 570 nm. The IC_50_ value, defined as the concentration of the extracts causing 50% cell growth inhibition, was determined from the cell survival diagrams.

#### 3.6.4. Morphological Analysis

The A375, LS-174, and MRC-5 cells were seeded into 96-well plates (Thermo Scientific Nunc™, Rochester, NY, USA) in nutrient medium. After 24 h of growth, the cells were treated with extracts in the desired range of concentrations. Following 72 h of treatment, the cells were observed under the inverted microscope for cell culture Olympus CKX53 (Tokyo, Japan) equipped with an Olympus EP50 camera (Tokyo, Japan), using a 10 × 0.25 objective.

## 4. Conclusions

Extracts of *A. ceterach* fronds exhibited significant antioxidant activity, moderate alpha-amylase inhibition, and notable antimicrobial and anti-biofilm effects without cytotoxic activity. The indicated biological activities may be attributed to the numerous polyphenolic compounds identified by untargeted metabolomic analysis, including phenolic acids and derivatives, flavonoid glycosides, and proanthocyanidins. These findings not only reinforce the traditional use of *A. ceterach* but also provide substantial evidence for the diverse therapeutic potential of this fern species, suggesting its promise as a source of natural compounds for managing oxidative stress and inflammation induced by microbial infections. Furthermore, the observed lack of cytotoxicity highlights the potential of *A. ceterach* extract as a safe matrix for developing novel phytotherapeutic preparations. Consequently, further research is required to elucidate the mechanisms of action of the *A. ceterach* extract and to develop novel therapeutic agents with antimicrobial and anti-biofilm activities. These findings represent a crucial screening step, while subsequent research focusing on optimizing extraction protocols using eco-friendly, safer solvents, alongside comprehensive stability and toxicological safety evaluations, is needed in order to facilitate future pharmaceutical applications.

## Figures and Tables

**Table 2:** Antibacterial and antifungal activity of *Asplenium ceterach* extracts. Minimum inhibitory concentration (MIC), bactericidal concentration (MBC), and fungicidal concentration (MFC) for fern extracts and standard compounds are given in mg mL^−1^. DCM—diclormethane extract of *A. ceterach*; ME—methanol extract of *A. ceterach*.

Species	DCM	ME	Standard
	MIC/MBC (mg mL^−1^)		
Gram (+) bacteria			Streptomycin
*Bacillus cereus*	0.50/1.00	0.25/0.75	0.006/0.012
*Staphylococcus aureus* ATCC 11632	1.50/2.00	0.50/1.00	0.05/0.10
*Listeria monocytogenes* NCTC 7973	1.50/2.00	1.00/2.00	0.05/0.10
Gram (−) bacteria			
*Escherichia coli* ATCC 25922	0.75/1.00	0.75/1.00	0.05/0.10
*Pseudomonas aeruginosa* ATCC 27853	1.50/2.00	1.50/2.00	0.05/0.10
*Salmonella Typhimurium* ATCC 13311	0.50/1.00	1.00/2.00	0.05/0.10
	MIC/MFC (mg mL^−1^)		
Fungi			Ketoconazole
*Aspergillus fumigatus* ATCC 204305	0.375/0.50	0.50/1.00	0.25/0.50
*Aspergillus niger* ATCC 6275	1.00/2.00	1.00/2.00	0.20/0.50
*Penicillium funiculosum* ATCC 36839	0.50/1.00	1.00/2.00	0.20/0.50
*Penicillium verrucosum* var. *cyclopium*	1.00/2.00	1.50/2.00	0.20/0.50
*Candida albicans* ATCC 10231	0.50/1.00	1.00/2.00	0.15/0.20
*Candida krusei*	0.25/1.00	1.00/2.00	0.05/0.10

**Table 3:** Antibiofilm activity of *Asplenium ceterach* extracts. Values represent the percentage inhibition of *Candida albicans* biofilm formation induced by fern extracts, given as mean ± SD of three replicates. DCM—dichloromethane extract of *A. ceterach*; ME—methanol extract of *A. ceterach*.

	MIC	0.5 MIC	0.25 MIC
DCM	74 ± 3	72 ± 7	48 ± 1
ME	73 ± 2	73 ± 2	20 ± 5
Ketoconazole	81 ± 14	73 ± 11	71 ± 12

## Data Availability

The original contributions presented in this study are included in the article/Supplementary Material. Further inquiries can be directed to the corresponding authors.

## Supplementary Materials

The following supporting information can be downloaded at https://www.mdpi.com/article/10.3390/molecules31172978/s1. Figure S1: Morphological changes in A375, LS-174 and MRC-5 cells after treatment with 100 µg mL^−1^ of ME and DCM extracts of *A. ceterach* for 72 h.

## Abbreviations

The following abbreviations are used in this manuscript:

ME	Methanol extract of *Asplenium ceterach*
DCM	Dichloromethane extract of *Asplenium ceterach*
DT	Desiccation-tolerant
HPTLC	High-performance thin-layer chromatography
CGA	5-*O*-caffeoylquinic acid
EPR	Electron Paramagnetic Resonance
DPPH	2,2-diphenyl-1-picrylhydrazyl
HRMS	High-Resolution Mass Spectrometry
MIC	minimum inhibitory concentrations
MBC	minimum bactericidal concentration
MFC	minimum fungicidal concentration
MTT	3-(4,5-dymethylthiazol-yl)-2,5-diphenyltetrazolium bromide
DMSO	dimethyl sulfoxide
